# The Spatial Representation of Self-Referential Negative Events

**DOI:** 10.3390/bs16091514

**Published:** 2026-08-28

**Authors:** Massimiliano Conson, Roberta Cecere, Camilla Maria Cleofe Puini, Francesco Panico, Isa Zappullo, Laura Sagliano, Luigi Trojano

**Affiliations:** Department of Psychology, University of Campania Luigi Vanvitelli, 81100 Caserta, Italy; roberta.cecere@unicampania.it (R.C.); camillamariacleofe.puini@unicampania.it (C.M.C.P.); francesco.panico@unicampania.it (F.P.); isa.zappullo@unicampania.it (I.Z.); laura.sagliano@unicampania.it (L.S.); luigi.trojano@unicampania.it (L.T.)

**Keywords:** spatial cognition, compatibility effect, rumination, worry, psychological distancing

## Abstract

The left-to-right spatial representation of time is reflected in faster responses when past-related concepts are associated with the left side of space and future-related concepts with the right. Most research has examined this time–space congruency effect using general temporal concepts, leaving unclear whether it also characterizes personally relevant temporal contents. The present study investigated whether the time–space congruency effect extends to self-referential negative events referring to the past and the future, and whether its expression is related to individual differences in maladaptive negative thinking. Forty-seven adults categorized individually generated words referring to past or future negative personal events, as well as neutral temporal words, by making left- or right-hand responses. A significant time–space congruency effect was observed for both self-referential and neutral words. The congruency effect was numerically smaller for self-referential words, and its strength was significantly and inversely related to anxiety sensitivity, whereas no such association was found for neutral words. These findings extend the mental timeline account to personally relevant negative events and provide initial evidence that individual differences in anxiety sensitivity are related to the spatial representation of such events. Psychological distance may represent a plausible mechanism underlying this association, a possibility that warrants direct testing in future research.

## 1. Introduction

Individuals from cultures with left-to-right writing systems mentally organize temporal information along a left-to-right spatial continuum, commonly referred to as the mental timeline, whereby the past is associated with left space and the future with right space (Tversky et al., 1991; Santiago et al., 2007, 2010; Torralbo et al., 2006; Ulrich & Maienborn, 2010). This spatial organization is consistently reflected in the time–space congruency effect; namely, responses are faster when past-related concepts require a left-hand response and future-related concepts require a right-hand response than when the opposite mapping is required (Santiago et al., 2007, 2010; Torralbo et al., 2006). Similar effects have been demonstrated using a wide variety of temporal stimuli and paradigms (e.g., Conson et al., 2008; Ishihara et al., 2008; Scozia et al., 2023, 2024; Vallesi et al., 2008), supporting the view that spatial coding constitutes a fundamental characteristic of temporal cognition. Despite the robustness of this phenomenon, previous studies have largely investigated the time–space congruency effect using general temporal concepts or conventional temporal expressions. Considerably less is known about whether the same effect can be found for temporally oriented representations that are self-referential and emotionally salient.

Spatial mapping of common life events has been demonstrated in studies on mental time travel, showing that people frequently relive past experiences and imagine possible future events by projecting themselves and the imagined events on a mental timeline (Anelli et al., 2016; Arzy et al., 2009; Buckner & Carroll, 2007; D’Argembeau & Van der Linden, 2004). Whether and how emotionally salient self-referential events, such as negative events that people often dwell on in daily life, are represented along the mental timeline remains poorly understood. Repetitive negative thinking provides an ideal framework for addressing this issue. Two of its most extensively investigated forms, rumination and worry, are characterized by repetitive negative self-referential thoughts predominantly oriented toward the past and the future, respectively (Borkovec et al., 1983; Ehring & Watkins, 2008; Nolen-Hoeksema et al., 2008). Although the distinction between rumination and worry is not sharp, as both may involve thoughts referring to past and future (Borkovec et al., 1998; Molina et al., 1998; Stade & Ruscio, 2023), temporal orientation remains one of their most distinctive characteristics (Ehring & Watkins, 2008; Watkins et al., 2005).

Negative events involved in repetitive negative thinking are typically experienced as closer in time to the present moment than emotionally neutral events (Bruehlman-Senecal & Ayduk, 2015; Siedlecka et al., 2015). Instead, neutral information encoded multiple times in a laboratory setting can be subsequently perceived as having initially occurred substantially further away in time (Sherman & Yousif, 2025). This apparent discrepancy may reflect the different nature of the repeated representations, with emotionally salient autobiographical events engaging mechanisms that differ from those involved in repeated exposure to neutral laboratory stimuli.

Repetitive negative thinking may represent a mechanism through which emotionally salient negative events remain psychologically close to the present, possibly reflecting attempts to achieve closure or to better understand their causes and consequences (Nolen-Hoeksema et al., 2008; Siedlecka et al., 2015). This raises the possibility that self-relevance and emotional salience may influence the spatial representation of temporal information through changes in perceived psychological proximity. Thus, rather than assuming that the mental timeline operates uniformly across different temporal contents, it is plausible that the spatial–temporal mapping varies depending on characteristics of the represented events such as self-relevance and emotional salience, and on individual differences in negative thinking.

The present study addressed this issue by testing the time–space congruency effect for negative self-referential events referring to the past and the future and comparing them with neutral temporal concepts. To ensure that the negative events were personally meaningful and emotionally salient for each participant, rather than relying on a standardized list of self-referential negative concepts, participants were asked to generate their own negative events. This approach allowed us to investigate representations that more closely reflect those involved in everyday repetitive negative thinking (Schlosser et al., 2011; Siegle et al., 2001). The participant-generated words referring to these events, together with words referring to neutral temporal concepts, were then used as stimuli in an experimental task previously employed to investigate the time–space congruency effect for general past- and future-related concepts (Santiago et al., 2007, 2010; Torralbo et al., 2006). Furthermore, individual differences in two dispositions associated with maladaptive negative thinking were assessed using two widely adopted self-report questionnaires: the Anxiety Sensitivity Index-3 (ASI-3; Taylor et al., 2007) and the Brooding subscale of the Ruminative Responses Scale (RRS; Nolen-Hoeksema & Morrow, 1991). The ASI-3 was selected because anxiety sensitivity has been consistently associated with maladaptive cognitive styles characterized by heightened attention to negative information and persistent negative thinking, and it shows strong associations with measures of both worry and rumination (Floyd et al., 2005; Ho et al., 2018; Klein et al., 2018; Yang et al., 2023). The Brooding subscale of the RRS was selected because brooding represents the maladaptive component of rumination and captures persistent, self-referential negative thinking (Ehring & Watkins, 2008).

We expected to observe a reliable time–space congruency effect for both self-referential negative and neutral temporal stimuli, indicating that even self-referential negative events are spatially organized according to their temporal orientation. However, the congruency effect might be weaker for self-referential words than for neutral temporal contents, possibly because self-relevant negative events are represented as closer in time to the present moment. Importantly, we further examined whether the strength of the time–space congruency effect for self-referential negative words was related to individual differences in maladaptive negative thinking. If greater engagement in maladaptive negative thinking is associated with increased psychological proximity to negative events, as suggested by previous research (Siedlecka et al., 2015), this should be reflected in a weaker time–space congruency effect for self-referential negative words with higher levels of maladaptive thinking. We did not expect such an association for neutral temporal contents.

## 2. Materials and Methods

### 2.1. Participants

To identify a proper target sample size, we conducted an a priori power analysis (G*power; Faul et al., 2007) to ensure detection of an effect of critical interest with a power of 0.80 at an alpha level of 0.05 with a medium effect size (f = 0.25) for a within-factor repeated-measures ANOVA. The analysis indicated that we needed at least 45 participants to detect an effect of critical interest for the ANOVA two-way interaction (time orientation x response side, as in the previous literature on time–space congruency effect for past- and future-related words; Santiago et al., 2007), setting the number of groups = 1, the number of measurements = 4, and r = 0, among the repeated measures, for a more conservative approach (Erle & Topolinski, 2017).

A sample of 47 right-handed University students (mean age = 23.82; SD = 2.84) was recruited on a voluntary basis. Individuals could participate if they were right-handed, as confirmed by their scores on the Edinburgh Handedness Inventory (Oldfield, 1971), and had no history of neurological or psychiatric conditions, or a diagnosis of neurodevelopmental disorder, and did not use substances acting on the central nervous system.

### 2.2. Procedure

Participants completed the study in person in two sessions. During the first session, after providing written informed consent, participants completed two self-report questionnaires and a word generation task. Five-to-seven days later, during the second session, they performed an experimental task assessing the time–space congruency effect.

#### 2.2.1. Self-Report Questionnaires

The Anxiety Sensitivity Index-3 (ASI-3; Taylor et al., 2007) measures the degree to which a person is concerned about possible negative consequences of anxiety symptoms. The ASI-3 includes three subscales: physical concerns, referring to the fear of somatic anxiety symptoms which are believed to lead to a catastrophic physical issue; social concerns, related to the belief that anxiety symptoms will manifest in a social context; and cognitive concerns, referring to the fear of the mental correlates of anxiety symptoms considered as signals of a mental health disorder. The ASI-3 total score corresponds to the sum of all the 18 items comprising the scale. In the present study, the ASI-3 total score derived from the Italian version of the questionnaire was adopted (Petrocchi et al., 2014).

The Ruminative Response Scale (RRS) is a subscale of the Response Style Questionnaire measuring trait rumination (Nolen-Hoeksema & Morrow, 1991). Treynor et al. (2003) analyzed the original, 22-item version of the scale (Nolen-Hoeksema & Morrow, 1991) and identified three factors: reflection, an adaptive way of thinking leading to effective problem-solving; brooding, a maladaptive way of thinking focused on comparison of one’s current situation with some unachieved standard; and depression, measuring depression-related symptoms. In the present study, the Italian version of the 22-item RRS, which has been proven to replicate the three-factor structure of the original version of the scale, was adopted (Palmieri et al., 2007). For the purpose of the research, only the Brooding score was used.

#### 2.2.2. Word Generation Task

Participants were first instructed to recall past or imagine future negative stressful events or experiences they thought about repeatedly and then generate words that best represented these events or experiences (Schlosser et al., 2011; Siegle et al., 2001). In detail, participants were first provided with the general instruction derived from studies on momentary assessment of worry and rumination (Baik & Newman, 2023, 2025). The instruction was as follows: “*I would like you to recall past negative stressful events or experiences you find yourself thinking about repeatedly and provide for each of them a word that best represents it. I ask you to do the same for possible future negative stressful events or experiences you find yourself thinking about repeatedly*”. After having received the general instruction, participants were provided with two separate sheets, labelled past and future, and asked to write down on each sheet 12 words that best corresponded to the mentally represented negative stressful events. Presentation of each sheet was preceded by a past-specific (“*I would like you to recall past negative stressful events or experiences…*”) or a future-specific (“*I would like you to imagine possible future negative stressful events or experiences…*”) instruction. For each list, after writing down the 12 words, participants were required to rank each word according to the degree of emotional intensity of the event it represented, assigning numbers in designated boxes ranging from 1 (most emotionally intense) to 12 (least emotional intense).

Participants were asked to generate 12 words for each sheet to ensure that at least 10 words could be selected from each list for the experimental task (see below): the 10 most highly ranked past-related words, and the 10 most highly ranked future-related words. The presentation order of the past and the future sheet was randomized across participants; immediately after having completed one list and the corresponding ranking, the participants were required to complete the second list. Participants completed the word generation task individually in a quiet room, free from distractions. The experimenter was present only to provide the instructions and clarifications when needed.

#### 2.2.3. Experimental Task

The stimuli consisted of 20 negative self-referential words (10 past-related and 10 future-related), individually generated by each participant during the word generation task, plus 20 neutral words (10 referring to the past and 10 referring to the future, matched for length and lexical frequency based on Bertinetto et al., 2005), primarily drawn from previous studies (Santiago et al., 2007; see Supplementary Materials for examples of participant-generated self-referential words and the complete list of neutral words). All stimuli were presented one at a time, horizontally and vertically centered on a PC monitor, in white using an 18-point monospaced font against a black background.

Participants were asked to decide, as quickly and accurately as possible, whether each word referred to the past or the future. Each trial began with a central fixation cross, presented for 500 ms, followed by a stimulus displayed at the center of the screen until a response was made. Responses were collected using the S and L keys of a standard QWERTY keyboard, pressed with participants’ left and right index fingers, respectively.

The task comprised two response-mapping conditions that differed in the spatial correspondence between response keys and word contents. In the spatially congruent condition, participants pressed the left key (S) for past and the right key (L) for future words, whereas in the spatially incongruent condition, the reverse mapping was used (S key for future; L key for past). The order of the congruent and incongruent response-mapping conditions was randomized across participants. Within each response-mapping condition, the 40 stimuli were presented in two separate blocks (consisting of 20 negative self-referential and 20 neutral words) randomized across participants. Thus, each participant completed a total of 80 trials, with each word presented once under the congruent mapping and once under the incongruent mapping.

Stimulus presentation and response recording (accuracy and Reaction Times, RTs) were performed using OpenSesame (version 4.0.5) on an HP Z2 G4 Workstation (Intel Core i7-9700 CPU @ 3.00 GHz, HP Inc., Palo Alto, CA, USA), which included an NVIDIA GeForce GT 710 graphics card (NVIDIA Corporation, Santa Clara, CA, USA). The PC monitor had a resolution of 1920 × 1080 pixels and a refresh rate of 60 Hz. The keyboard was centered on the table, at a constant viewing distance of about 60 cm. Participants were instructed to maintain a comfortable sitting position. The task was administered in a darkened room to help participants to maintain their attention on the screen and minimize external distractions. At the end of the task, they were fully debriefed and informed about the true purpose of the study.

### 2.3. Statistical Analysis

Accuracy and RTs were recorded. Following previous studies (Santiago et al., 2007, 2010), RT analyses focused on correct responses only. Incorrect responses were excluded, and, among the remaining correct responses, RTs exceeding 2.5 standard deviations from each participant’s mean RTs were further excluded as outliers (2.35% of correct responses). After these exclusions, participants contributed an average of 15.51 (SD = 4.71) trials to the neutral–incongruent cell, 17.94 (SD = 1.62) to the neutral–congruent cell, 14.09 (SD = 3.60) to the self-referential–incongruent cell, and 14.89 (SD = 2.96) to the self-referential–congruent cell. RTs were then submitted to a 2 × 2 × 2 repeated-measures ANOVA, with time orientation (past vs. future), response side (left vs. right) and word content (self-referential vs. neutral) as within-subject factors. Post hoc Bonferroni’s corrected pairwise comparisons were performed when needed.

To provide a further direct test of the time–space congruency effect and determine whether its strength varied as a function of word content, dRTs were calculated by subtracting RTs for congruent trials from RTs for incongruent trials for each participant, separately for self-referential and neutral words. Positive dRTs values indicate a time–space congruency effect, with larger positive values indicating a stronger effect. dRTs for self-referential and neutral words were compared using a paired-samples *t*-test, and dRTs for each word-content condition were separately tested against zero using one-sample *t*-tests.

Finally, to evaluate whether the strength of the time–space congruency effect was related to interindividual differences in maladaptive negative thinking, Pearson correlations were conducted between dRTs for self-referential and neutral words, and the Z-scores on the ASI-3 and the Brooding subscale of the RRS.

## 3. Results

**Figure 1:**
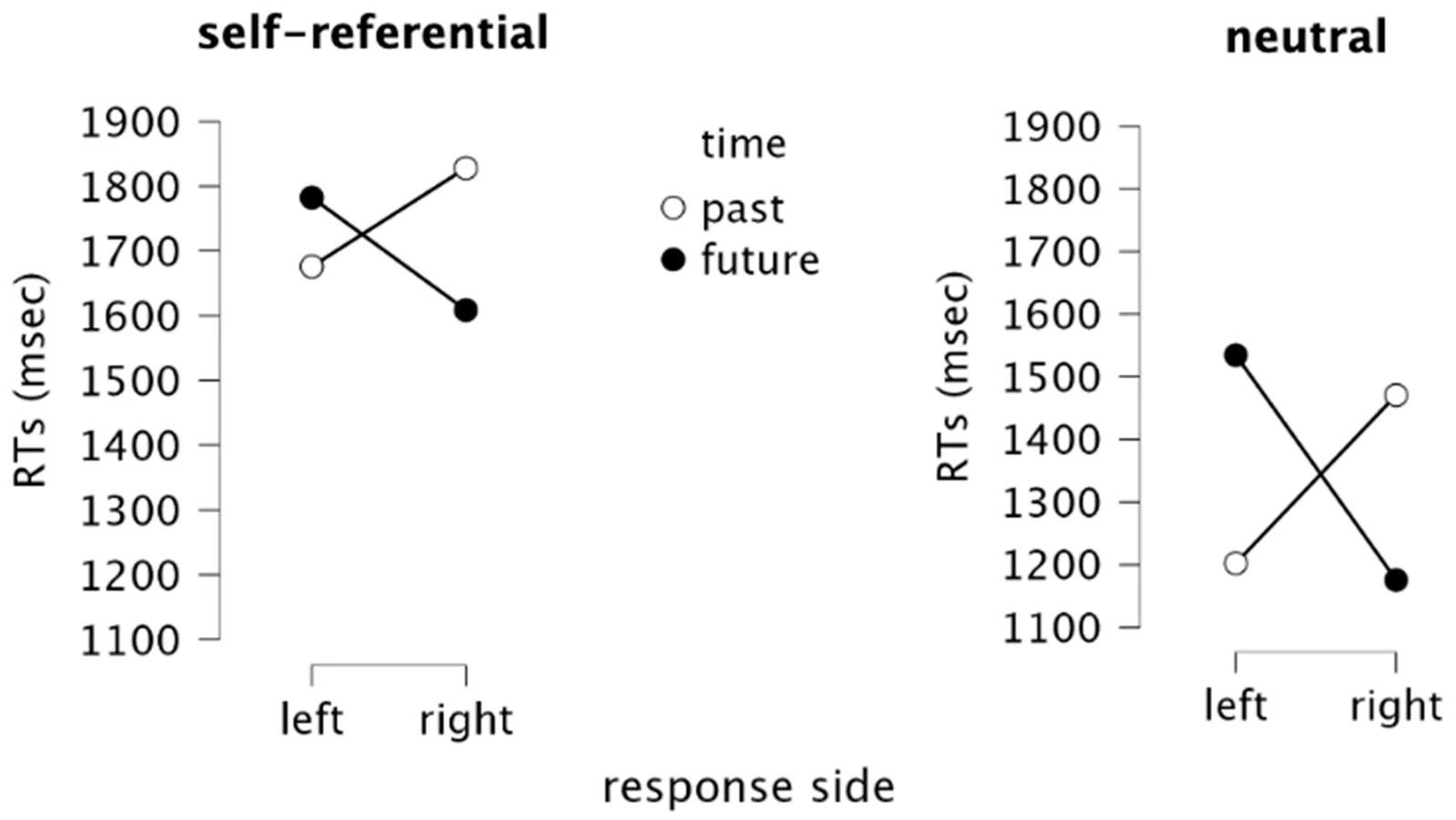
Mean RTs of correct responses to self-referential (**left panel**) and neutral (**right panel**) past and future words as a function of response side.

Results of the ANOVA showed a significant main effect of word content, F(1,46) = 19.744, *p* < 0.001, η^2^ = 0.119, with slower responses to self-referential (M = 1723, SEM = 98.9) than to neutral words (M = 1346, SEM = 78.8). A significant interaction was also found between time orientation and response side, F(1,46) = 24.885, *p* < 0.001, η^2^ = 0.048. Responses to past words were faster with the left than with the right key (t = −2.797, *p* = 0.045), whereas responses to future words were faster with the right than with the left key (t = 4.304, *p* < 0.001), consistent with the time–space congruency effect. The three-way interaction among time orientation, response side and word content was not significant (*p* = 0.198; Figure 1). No other F was significant at the 0.05 level.

dRTs showed more positive values for neutral (mean = 313, SEM = 69.2) than for self-referential words (mean = 163, SEM = 80.1), although this difference was not statistically significant in a paired-samples *t*-test (t = −1.306, *p* > 0.05). However, one-sample *t*-tests showed that dRTs for self-referential words were significantly greater than zero (t = 2.034, *p* = 0.048), as were dRTs for neutral words (t = 4.530, *p* < 0.001). Thus, the time–space congruency effect was evident for both self-referential and neutral words, with a numerically smaller effect for self-referential than for neutral words, although the difference between the two dRTs did not reach statistical significance.

**Figure 2:**
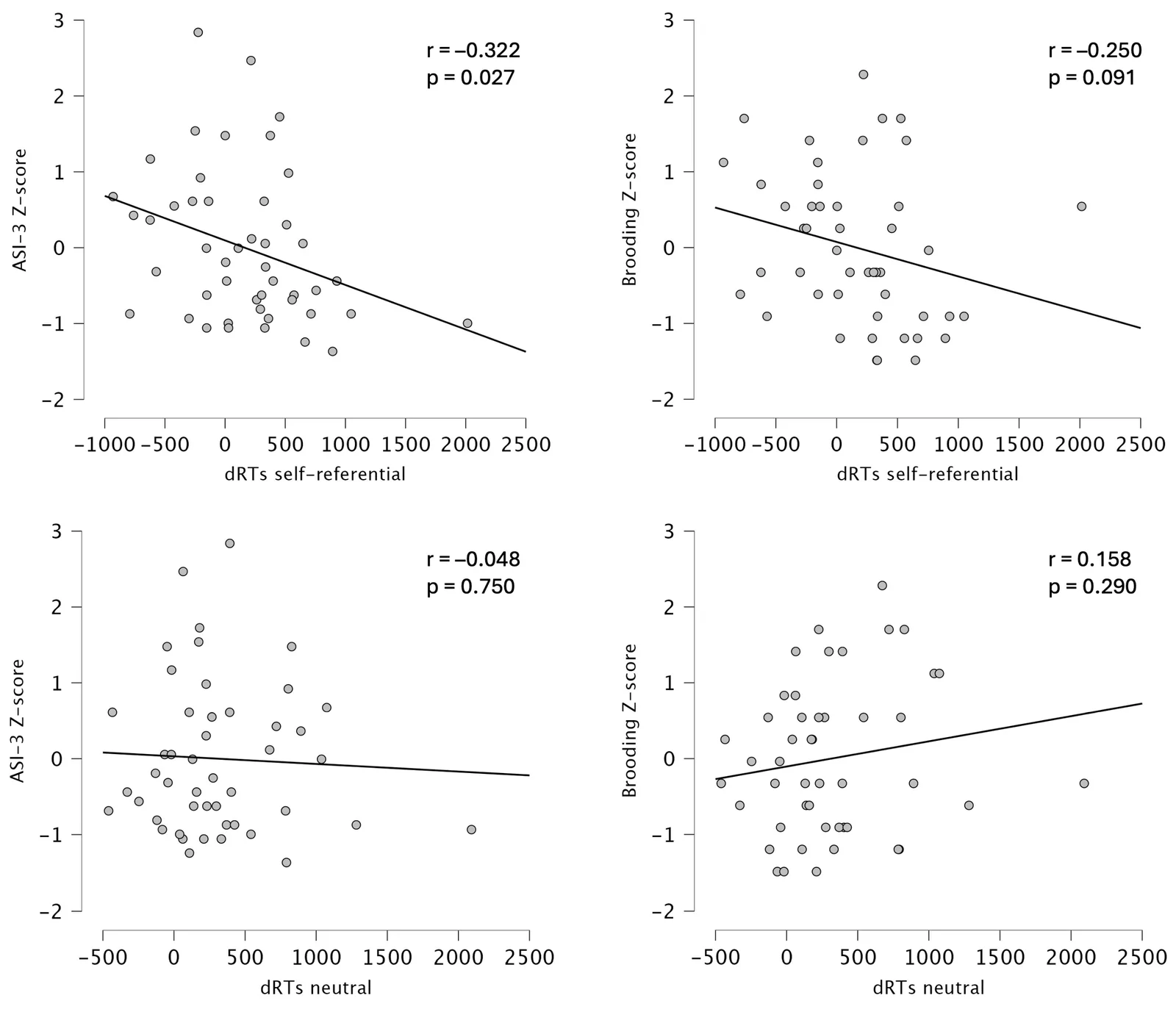
Plots of the Pearson correlation coefficients between dRTs for self-referential (**upper panels**) and neutral (**lower panels**) words, and the Z-scores on the ASI-3 and the Brooding subscale of RRS.

Pearson correlation analyses showed a significant negative correlation between dRTs for self-referential words and ASI-3 score (r = −0.322, *p* = 0.027), whereas the negative correlation between dRTs and the Brooding score of the RRS did not reach statistical significance (r = −0.250, *p* = 0.091). In contrast, dRTs for neutral words were not significantly correlated with either ASI-3 (r = −0.048, *p* = 0.750) or Brooding scores (r = 0.158, *p* = 0.290; Figure 2). Thus, the strength of the time–space congruency effect for self-referential words, but not neutral words, was significantly associated with individual differences in anxiety sensitivity, with higher ASI-3 score being associated with a weaker congruency effect.

## 4. Discussion

The present study investigated whether the left-to-right spatial representation of time extends beyond abstract temporal concepts to personally relevant negative events. The results showed the canonical time–space congruency effect for both self-referential and neutral stimuli, although it was numerically smaller for self-referential than for neutral words.

A broad debate has concerned similarities and differences between two main types of repetitive negative thoughts, that is, worry and rumination (e.g., Fresco et al., 2002; Hur et al., 2017; McEvoy et al., 2013; Segerstrom et al., 2000; Stade & Ruscio, 2023; Watkins et al., 2005). More similarities than differences have been found between these two phenomena, supporting the view that both share common processes while differing, among other features, in the temporal orientation of their contents (Ehring & Watkins, 2008; Hur et al., 2017; Papageorgiou & Wells, 1999; Watkins et al., 2005). The present results do not contribute to the issue of whether rumination and worry are different or overlapping processes (Stade & Ruscio, 2023), but indicate that self-referential negative contents oriented towards the past or future can be spatially distinguished along a left-to-right mental timeline, with past events associated with the left side and future events with the right side.

Importantly, the strength of the time–space congruency effect for self-referential words was inversely related to anxiety sensitivity, whereas no significant association was found for neutral words. This finding suggests that individual differences in maladaptive negative thinking may be particularly relevant to the spatial representation of personally relevant negative events.

Self-referential events related to repetitive negative thoughts are mentally represented as closer in time to the present moment (Siedlecka et al., 2015; see also, Bruehlman-Senecal & Ayduk, 2015). Moreover, the time–space congruency effect is reduced when events to be spatially mapped are closer to a given reference point (Santiago et al., 2010). Against this background, the finding that participants with a higher ASI-3 score showed a weaker congruency effect raises the possibility that the spatial representation of personally relevant negative events may be related to their perceived psychological proximity.

The mechanisms linking repetitive negative thinking to perceived temporal closeness, however, remain unclear, and neither the present findings nor the available literature provide a definitive explanation (Siedlecka et al., 2015). The effects of repetition on subjective temporal experience may depend on the nature of the repeated representations, since emotionally salient autobiographical events are experienced as temporally closer to the present (Siedlecka et al., 2015), whereas repeated neutral information may be remembered as more temporally distant (Sherman & Yousif, 2025). Insights into these mechanisms may come from research on psychological distancing (Ayduk & Kross, 2010; Bruehlman-Senecal & Ayduk, 2015; Kross, 2009; Kross & Ayduk, 2011). Temporal distance from an experienced or imagined event influences the representation of its phenomenal characteristics, such that temporally close events include more sensorial and contextual details, elicit stronger feelings of re-experiencing or pre-experiencing, and produce a greater emotional impact than temporally distant events (Bruehlman-Senecal & Ayduk, 2015; D’Argembeau & Van der Linden, 2004). Conversely, greater psychological distance from a negative event is associated with lower perceived threat and better regulation of the related emotional response (Bruehlman-Senecal & Ayduk, 2015; Powers & LaBar, 2019). Also, distancing may reduce the intensity of emotional experiences and related maladaptive cognitive phenomena like repetitive negative thinking (Ayduk & Kross, 2010; Kross, 2009; Kross & Ayduk, 2011). Because people reliving past experiences and imagining possible future events (Buckner & Carroll, 2007; D’Argembeau & Van der Linden, 2004) project themselves and the imagined events along a mental timeline (Arzy et al., 2009), one possibility is that events perceived as more threatening are represented as psychologically closer to the self along this timeline, potentially reducing the strength of the spatial–temporal mapping. In this context, the numerically smaller dRTs observed for self-referential words, together with their inverse association with anxiety sensitivity, may be consistent with the possibility that the strength of the spatial–temporal mapping reflects, at least in part, the perceived psychological proximity of personally relevant negative events.

Some limitations of the present study should be acknowledged. The first concerns the use of participant-generated self-referential events, while the neutral words referred to general temporal concepts. This procedure ensured that the stimuli were personally meaningful and emotionally salient for each participant, but it may also have increased participants’ familiarity with these stimuli relative to the standardized neutral words. Furthermore, because the experimental task was administered 5–7 days after the word-generation session, participants may have continued to think about some of the generated events during the interval. Although familiarity or rehearsal effects may have influenced overall response times, they are unlikely to fully account for the time–space congruency effect observed in the present study. Future studies could address this issue by matching self-referential and neutral stimuli for familiarity, or by including standardized self-referential stimuli as a comparison condition. Such designs could also benefit from trial-level mixed-effects models to account for variance associated with both participants and items. Second, we did not require participants to estimate the temporal distance of the events corresponding to the self-referential words selected for the experimental task. Because perceived temporal distance was not assessed, these aspects prevented us from directly testing whether the strength of the congruency effect varied as a function of the perceived temporal distance of individual events. Thus, further work overcoming these limitations is warranted. Additional support to the present interpretation could come from experimental studies manipulating temporal distancing (Bruehlman-Senecal & Ayduk, 2015; Kross & Ayduk, 2011, 2017). In this perspective, it could be possible to test whether experimentally increasing or reducing the temporal distance from a self-referential negative event produces corresponding changes in the strength of the time–space congruency effect.

## 5. Conclusions

The present study shows that the mental timeline extends to personally relevant negative events and provides initial evidence that individual differences in anxiety sensitivity are related to the spatial representation of such events. The time–space congruency effect was evident for both self-referential and neutral temporal contents, while greater anxiety sensitivity was associated with a weaker congruency effect for self-referential negative events only. Psychological distance may provide a potential mechanism underlying this association, but future research should test this hypothesis directly.

## Data Availability

The data and materials for the present research are available at https://osf.io/dtk97/files/4vs2f (accessed on 3 February 2026).

## Supplementary Materials

The following supporting information can be downloaded at: https://www.mdpi.com/article/10.3390/bs16091514/s1, Table S1: Examples of participant-generated self-referential words referring to personally relevant negative events in the past, the future, or both temporal orientations across participants; Table S2: Complete stimulus list of neutral words.
