# Supplementary material for: The Spatial Representation of Self-Referential Negative Events
Source: Behav Sci (Basel). 2026 Aug 28;16(9):1514. doi: 10.3390/bs16091514 (PMC13603467; doi:10.3390/bs16091514)
Supplement: Supplementary file 1 [file behavsci-16-01514-s001.zip › behavsci-4453373-supplementary.pdf]

Supplementary Materials

Participants generated a total of 395 unique self-referential words to represent negative events referring to the past and future. Some words were generated more frequently than others, as the same word could be generated by multiple participants. Moreover, some words were classified as past-related by some participants and as future-related by others, whereas other words were consistently classified as either past- or future-related. Given the large number and variability of participant-generated words, Table S1 provides illustrative examples of self-referential words used to represent past- and future-oriented negative events.

Table S1. Examples of participant-generated self-referential words (presented in Italian, with English translations in brackets) referring to personally relevant negative events in the past, the future, or both temporal orientations across participants.

Temporal orientation	Examples
Past	*bullismo (bullying)*, *bugie* (lies), *cibo* (food), *dolore* (pain), *esclusione* (exclusion), *incidente* (accident), *lacrime* (tears), *litigio* (quarrel), *rifiuto* (rejection), *silenzio* (silence)
Future	*felicità* (happiness), *figli* (children), *incertezza* (uncertainty), *laurea* (degree), *lavoro* (work), *libertà* (freedom), *ripudio* (repudiation), *salute* (health), *soldi* (money), *stabilità* (stability)
Past and Future across participants	*amore* (love), *casa* (home), *esame* (exam), *famiglia* (family), *lutto* (mourning), *mamma* (mom), *morte* (death), *ospedale* (hospital), *relazioni* (relationships), *università* (university)

Table S2 provides the complete list of 20 neutral Italian words: 10 referring to the past and 10 referring to the future, following the classification criteria used in Santiago et al.'s (2007) study. Some words were directly selected from Santiago et al. (2007), while the remaining ones were selected to complete past and future lists while preserving the same linguistic constraints (temporal reference and neutrality). The past and future lists were matched on length (letters) and lexical frequency (CoLFIS: Bertinetto et al., 2005). Descriptives indicated a close matching between the two lists (length: past M = 6.80, SD = 1.93; future M = 7.00, SD = 2.06; frequency: past M = 805.10, SD = 1481.59; future M = 754.20, SD = 1576.33). Independent-samples t-tests showed no significant differences between past- and future-related words in either length, $t(18) = -0.224$, $p = .825$, or lexical frequency, $t(18) = 0.074$, $p = .942$, confirming comparable matching between the two lists (see Table S2).

Table S2. Complete stimulus list neutral words in Italian (with English translations in brackets).

Past	Future
andai (I went)	*andrò* (I will go)
anteriore (previous)	*berrò* (I will drink)
bevvi (I drank)	*davanti* (ahead)
ieri (yesterday)	*dopo* (after)
indietro (back)	*futuro* (future)
mangiai (I ate)	*mangerò* (I will eat)
passato (past)	*posteriore* (posterior)
precedere (to precede)	*proseguire* (to proceed)
prima (before)	*prossimo* (next)
trascorso (elapsed)	*seguente* (following)

References

Bertinetto, P. M., Burani, C., Laudanna, A., Marconi, L., Ratti, D., Rolando, C., & Thornton, A. M. (2005). *Corpus e Lessico di Frequenza dell'Italiano Scritto (CoLFIS).* http://linguistica.sns.it/CoLFIS/CoLFIS_home.htm.

Santiago, J., Lupiáñez, J., Pérez, E., & Funes, M. J. (2007). Time (also) flies from left to right. *Psychonomic Bulletin & Review*, 14(3), 512–516.
